# Transgenic CD8αβ co-receptor rescues endogenous TCR function in TCR-transgenic virus-specific T cells

**DOI:** 10.1136/jitc-2020-001487

**Published:** 2020-11-03

**Authors:** Gagan Bajwa, Inès Lanz, Mara Cardenas, Malcolm K Brenner, Caroline Arber

**Affiliations:** 1 Department of Oncology UNIL CHUV, Lausanne University Hospital, Ludwig Institute for Cancer Research, University of Lausanne, Lausanne, Switzerland; 2 Center for Cell and Gene Therapy, Baylor College of Medicine, Houston, Texas, USA

**Keywords:** cell engineering, immunotherapy, adoptive, receptors, antigen

## Abstract

**Background:**

Genetically engineered virus-specific T cells (VSTs) are a platform for adoptive cell therapy after allogeneic hematopoietic stem cell transplantation. However, redirection to a tumor-associated antigen by the introduction of a transgenic T-cell receptor (TCR) reduces anti-viral activity, thereby impeding the possibility of preventing or treating two distinct complications—malignant relapse and viral infection—with a single cell therapy product. Availability of CD8αβ co-receptor molecules can significantly impact class I restricted T-cell activation, and thus, we interrogated whether transgenic CD8αβ improves anti-viral activity mediated by native VSTs with or without a co-expressed transgenic TCR (TCR8).

**Methods:**

Our existing clinical VST manufacturing platform was adapted and validated to engineer TCR+ or TCR8+ VSTs targeting cytomegalovirus and Epstein-Barr virus. Simultaneous anti-viral and anti-tumor function of engineered VSTs was assessed in vitro and in vivo. We used pentamer staining, interferon (IFN)-γ enzyme-linked immunospot (ELISpot), intracellular cytokine staining (ICS), cytotoxicity assays, co-cultures, and cytokine secretion assays for the in vitro characterization. The in vivo anti-tumor function was assessed in a leukemia xenograft mouse model.

**Results:**

Both transgenic CD8αβ alone and TCR8 had significant impact on the anti-viral function of engineered VSTs, and TCR8+ VSTs had comparable anti-viral activity as non-engineered VSTs as determined by IFN-γ ELISpot, ICS and cytotoxicity assays. TCR8-engineered VSTs had improved anti-tumor function and greater effector cytokine production in vitro, as well as enhanced anti-tumor function against leukemia xenografts in mice.

**Conclusion:**

Incorporation of transgenic CD8αβ into vectors for TCR-targetable antigens preserves anti-viral activity of TCR transgenic VSTs while simultaneously supporting tumor-directed activity mediated by a transgenic TCR. Our approach may provide clinical benefit in preventing and treating viral infections and malignant relapse post-transplant.

## Introduction

Malignant relapse and viral infections are the two major causes for treatment failure and morbidity in patients after allogeneic hematopoietic stem cell transplantation (HSCT).^1^ An ideal cellular therapy after stem cell transplant would therefore target both problems simultaneously. Virus-specific T cells (VSTs) are already a clinically validated immune effector cell therapy platform amenable to genetic redirection of antigen-specificity to tumor-associated antigens, as demonstrated with chimeric antigen receptor (CAR)-modified VST cell therapies.^2–6^ CAR+ VSTs can significantly re-expand in vivo upon viral reactivation and stimulation through the endogenous T-cell receptor (TCR) and persist long-term.^6^

Efforts to redirect VSTs to tumor by introduction of a transgenic TCR,^7–11^ however, have been more problematic. Forced expression of a transgenic TCR leads to downregulation of endogenous TCRs^12^ and consequent reduction in anti-viral reactivity, although anti-tumor activity can be sustained.^7–11^ The reduction in anti-viral activity was consistent across several studies by independent groups, using a variety of different VST platforms, TCR specificities and vectors. Anti-tumor function predominated consistently,^10 11^ or reactivities shifted between compartments depending on the type of antigen encountered (viral versus tumor antigen).^11^ These effects are most likely explained by competition for TCR/CD3/CD8 complex signaling components used by both the endogenous anti-viral and introduced transgenic TCRs, as well as TCR mis-pairing between introduced and endogenous TCR chains,^11–13^ and imply two important points: (i) the clinical benefit from controlling viral reactivation post transplant may be jeopardized when using TCR+ VSTs, and (ii) the capacity of TCR+ VSTs to re-expand in vivo upon viral reactivation or vaccination may be limited compared to CAR+ VSTs.

Incorporation of CD8αβ into the transgenic TCR vector enhances the function of polyclonal TCR+ T cells through multiple pathways,^14^ and here we investigated if this strategy rescues endogenous class I restricted anti-viral TCR function. We used a CD8-dependent TCR targeting the tumor-associated antigen survivin in the context of HLA-A*02:01 and expressed the TCR alone (TCR)^15^ or in combination with CD8αβ (TCR8)^14^ in VSTs targeting cytomegalovirus (CMV) and Epstein-Barr virus (EBV). We consistently generated TCR+ and TCR8+ VSTs with a predominant central memory phenotype and showed that anti-viral reactivities were restored in TCR8+ VSTs while anti-tumor function was retained.

## Materials and methods

### Cell lines

BV173 and K562 cell lines were obtained from the German Cell Culture Collection (DSMZ) and the American Type Culture Collection (ATCC), respectively, and maintained in complete RPMI 1640 media (Hyclone; Thermo Scientific) supplemented with 10% or 20% fetal bovine serum (FBS, Hyclone), 1% penicillin-streptomycin (Gibco), and 1% glutamax (Gibco). Two hundred and ninety-three T cells (ATCC) were maintained in complete IMDM media (Hyclone) containing 10% FBS, 1% penicillin-streptomycin, and 1% glutamax. For the mouse xenograft experiments, the previously described BV173.ffLuc cell line was used.^15^

### Blood samples from healthy donors

Buffy coats were obtained from CMV seropositive de-identified healthy human volunteers at the Gulf Coast Regional Blood Center (Houston, Texas, USA). HLA-A2 status was assessed by fluorescence-activated cell sorting (FACS) analysis and HLA-A2+ donors were selected for the experiments.

### Generation of retroviral vectors and supernatants

**Figure 1:**
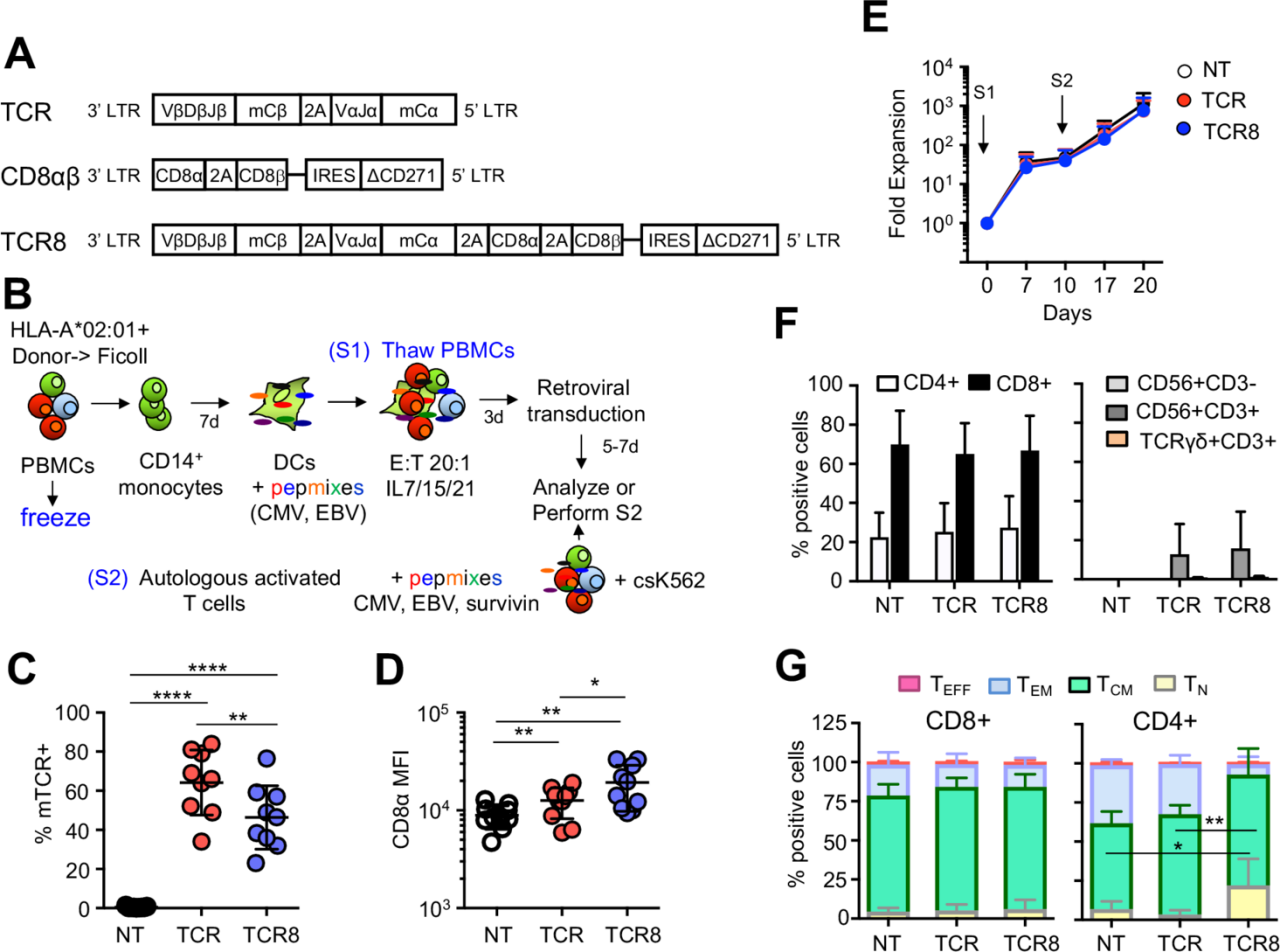
Generation and characterization of TCR+ and TCR8+ VSTs. (A) Schemes of retroviral vectors used in the study. (B) Scheme of transgenic VST production. S1: first stimulation, S2: second stimulation. (C) Transduction efficiencies of VSTs with TCR (red dots) or TCR8 (blue dots) vectors compared with NT controls (gated on live cells, %mTCR+ cells, n=9, mean±SD). (D) CD8α mean fluorescence intensity in VSTs (n=9, mean±SD). (E) Fold expansion of NT (white), TCR+ (red) and TCR8+ (blue) VSTs after S1 and S2 (n=9, mean±SD, p=ns). (F) Distribution of CD4+, CD8+, NK (CD56+ CD3−) or TCRγδ (TCRγδ+ CD3+) cells in NT, TCR+ and TCR8+ VSTs (n=5, mean±SD, p=ns). (G) Memory phenotype: T_N_, T_CM_, T_EM_, and T_EFF_ subset distribution in NT or transduced VSTs based on two-marker CD45RO/CD62L gating (n=5, mean±SD, one-way analysis of variance). CD4+ T_N_: NT versus TCR8+: *p=0.03; TCR+ versus TCR8+: **p=0.008. CD4+ T_CM_: NT versus TCR8+: *p=0.03; TCR+ versus TCR8+: p=ns. (A to F) Coding of significance levels: *p<0.05, **p<0.01, ***p<0.001, ****p<0.0001. CMV, cytomegalovirus; DC, dendritic cell; EBV, Epstein-Barr virus; NT, non-transduced; PBMC, peripheral blood mononuclear cell; TCR, T-cell receptor; VSTs, virus-specific T cells.

The retroviral vectors expressing the survivin-specific (s24) TCR and the combination of TCR and CD8αβ have previously been described.^14 15^ Genes encoding for the human CD8α (Uniprot P01732) and CD8β isoform 1 (βM1, Uniprot P10966-1) chains, separated by a 2A sequence, were synthesized by Geneart (Invitrogen) and cloned into the SFG retroviral vector backbone (figure 1A) (In-Fusion HD Cloning Kit, Clontech). Transient retroviral supernatants were prepared by co-transfection of 293 T cells with RD114 and Pegpam plasmids and the SFG vector containing the gene of interest.^16^

### Generation of gene-modified virus-specific T cells

Peripheral blood mononuclear cells (PBMCs) were isolated from buffy coats using density gradient centrifugation by Lymphoprep (Accurate Chemical and Scientific Corporation). CD14+ monocytes were positively selected from PBMCs with microbeads (Miltenyi Biotech) and grown in serum-free CellGenix dendritic cell (DC) media (CellGenix) supplemented with 1000 U/mL of Interleukin (IL) 4 and granulocyte-macrophage colony-stimulating factor (GM-CSF) each (Miltenyi) for 5 to 7 days to generate immature DCs. DCs were matured using a cocktail of cytokines, including IL4 (1000 U/mL), GM-CSF (800 U/mL), IL1β (10 ng/mL), tumor necrosis factor (TNF)-α (10 ng/mL), IL6 (100 ng/mL), and prostaglandin E2 (1 µg/mL), all from R&D Systems for 48 hours. For antigen pulsing, mature DCs were incubated for 60 min at 37°C with a mixture of CMV and EBV pepmixes (CMV pp65, CMV IE1, EBV LMP2, EBV BZLF1, EBV EBNA 1, all from JPT Technologies; 1 µg/mL), and HLA-A*02:01-restricted immunodominant peptides (CMV pp65-derived NLV (NLVPMVATV, 1 µg/mL), immediate early EBV BRLF1-derived YVL (YVLDHLIVV, 1 µg/mL), early EBV BMLF1-derived GLC (GLCTLVAML, 1 µg/mL), all from Genemed Synthesis.^17^ The combination of pepmixes and peptides was chosen based on antigen expression patterns of CMV and EBV infection in the post transplant period.^18 19^ Autologous PBMCs were thawed and stimulated with pepmix/peptide-pulsed mature DCs (E:T ratio 20:1) in T-cell media (1:1 mixture of RPMI 1640 and Click’s media, Hyclone), supplemented with 10% FBS (Hyclone), 1% penicillin-streptomycin, 1% glutamax and cytokines IL7 (10 ng/mL, R&D Systems), IL15 (10 ng/mL, R&D Systems), and IL21 (30 ng/mL, R&D Systems). On day 3, antigen-primed cells were harvested and retrovirally transduced for 2 to 3 days with the retroviral supernatants for TCR, TCR8, or CD8αβ on retronectin-coated plates or exposed to retronectin-coated plates without viral particles (for non-transduced VSTs). After transduction, cells were further expanded for 7 to 9 days in culture flasks or GRex gas permeable culture devices (Wilson Wolf, St. Paul, USA). A second stimulation was performed with pepmix/peptide-pulsed irradiated (40 Gy) autologous activated T cells (previously activated on OKT3/anti-CD28-coated plates) and irradiated (100 Gy) K562cs cells (K562 cells engineered to express CD80, CD83, CD86, 41BBL, and CD32) at a ratio of 1:1:5 as previously described,^20^ in T-cell media and cytokines IL7 (10 ng/mL), IL15 (10 ng/mL), and IL21 (30 ng/mL). For the second stimulation, the HLA-A*02:01-restricted survivin peptide LMLGEFLKL (Genemed Synthesis) that is recognized by the transgenic TCR was also included in the mixture. The method is good manufacturing practice (GMP) compatible.

### Immunophenotyping

Cell surface stainings were performed with fluorescein isothiocyanate (FITC), phycoerythrin (PE), allophycocyanin (APC), V450, PerCP, APC-AF750, or krome orange-conjugated antibodies against CD4, CD8, CD45RO, CD62L, CCR7, CD45RA, CD56, TCR γδ, CD271, CD19, 7-AAD (BD Biosciences), murine TCRβ constant region (eBioscience), survivin dextramer (Immudex), or NLV pentamer (Proimmune) for 30 min at 4°C. For the degranulation assay, VSTs (10^6^) were treated with golgiplug/brefeldin A (Invitrogen) and CD107a/b VioBlue (BD Biosciences) followed by appropriate stimulations: CMV/EBV-specific viral pepmixes or single peptide (pp65, IE1, LMP2, BZLF1, EBNA1, and GLC), survivin-specific LML peptide, viral pepmixes/peptide plus LML peptide, PMA (25 ng/mL)/ionomycin (1 µg/mL), or control influenza matrix protein GIL (GILGFVFTL, Genemed Synthesis) peptide (negative control) in T-cell media for 4 hours at 37°C. Intracellular staining was performed using anti-human interferon (IFN)-γ-FITC and TNF-α-PE (BD Biosciences). All samples were acquired on a FACS Canto with BD FACSDiva, and analysis was performed using FlowJo software (Tree Star).

### IFN-γ ELISpot

For the IFN-γ enzyme-linked immunospot (ELISpot) assay, 10^5^ VSTs per well were plated in triplicates and stimulated with individual pepmixes/peptides (1 µg/mL) or cell lines (BV173 or K562) at 1:1 ratio (10^5^ cells per well) or media alone. Plates were incubated at 37 °C/5% CO_2_ overnight and developed as previously described.^15^ Spot forming units were enumerated by ZellNet.

### Co-culture assay and cytokine detection

VSTs and BV173 cells were co-cultured at E:T ratio 1:5 in the absence of exogenous cytokines. Co-culture supernatants were harvested 24 hours after plating and stored at −80°C for cytokine analysis. Residual VSTs and tumor cells were enumerated after 3 days using CountBright Beads (Life Technologies) and FACS analysis. Cytokines were quantified in supernatants using the MILLIPLEX Human CD8+ T cells magnetic beads panel (EMD Millipore) and a Luminex 200 instrument (Luminex).

### ^51^Chromium release assay

To assess the short term in vitro cytotoxic function of VSTs, a standard ^51^Chromium (^51^Cr) release assay was performed.^15^ Autologous activated T cells were used as targets, pulsed with the indicated peptides or pepmixes and labeled with ^51^Cr for 1 hour. Effector and target cells were incubated at various ratios. As controls, target cells were incubated in media alone or with 1% Triton-X 100 (Sigma-Aldrich) for 4 hours to determine the spontaneous and the maximum release. The mean percentage of specific lysis of triplicate wells was calculated as follows: ((test counts – spontaneous counts)/(maximum counts − spontaneous counts))×100%.

### Mouse xenograft model

Female NOD-SCID-γc^−/−^ (NSG) mice (6 to 8 weeks old) were purchased from the Jackson Laboratory and housed at the Baylor College of Medicine Animal Facility. Mice were irradiated with 120 cGy and infused with 3×10^6^ BV173.ffLuc cells/mouse through the tail vein 4 to 6 hours later. Leukemia burden was monitored by bioluminescent imaging (BLI) (photons/s/cm^2^/sr) using the Xenogen in vivo imaging system (Caliper Life Sciences). Two VST injections (2×10^6^/mouse, 3 days apart) were administered through the tail vein or the retro-orbital vein plexus beginning 24 hours after tumor injection. Leukemia growth was monitored weekly by BLI and survival recorded.

### Statistical analysis

Data were summarized using descriptive statistics. Comparisons between groups were made using student’s t-test, one-way or two-way analysis of variance, and Friedman or Wilcoxon test, whichever was appropriate (figure legends). Area under the curves of bioluminescent signal intensity were calculated in GraphPad Prism from day 0 to day 28 and differences between groups were analyzed with the Mann-Whitney test. Survival of mice was analyzed by Kaplan-Meier graphs and differences were analyzed with the log-rank test. GraphPad prism 6 (GraphPad software, La Jolla, California) or higher was used for statistical analysis. P values <0.05 were considered statistically significant.

### Study approval

Animal studies were reviewed and approved by the Institutional Animal Care and Use Committee of Baylor College of Medicine.

## Results

### Generation of TCR+ and TCR8+ transgenic VSTs with central memory phenotype

We used three retroviral constructs encoding for the TCR alone, CD8αβ alone, or the combination of TCR and CD8αβ (TCR8) (figure 1A). PBMCs from CMV-seropositive HLA-A*02:01+ donors were used to generate mature DCs, pulsed with CMV and EBV pepmixes, and used to stimulate autologous PBMCs in the presence of cytokines. Three days later, activated T cells were retrovirally transduced and further expanded for an additional 5 to 7 days. A second stimulation of VSTs was performed if necessary, to further expand VSTs using pepmix pulsed autologous activated polyclonal T cells and irradiated csK562 cells (engineered to express several co-stimulatory molecules) (figure 1B and Methods). We achieved high transduction efficiencies with both TCR and TCR8 vectors. As expected, TCR8 transduction was less efficient because of the larger vector size (%mTCR+ cells, TCR+ versus TCR8+: 64±17% vs 46±16%, **p=0.002, mean±SD, n=9) (figure 1C). We detected higher CD8α mean fluorescence intensity (MFI) in VSTs transduced with TCR8 compared with TCR or NT VSTs (CD8α MFI NT versus TCR+: 8.9±2.4×10^3^ vs 12.6±4.4×10^3^, **p=0.002, TCR+ versus TCR8+: 12.6±4.4×10^3^ vs 19.3±9.5×10^3^, *p=0.03, NT versus TCR8+: 8.9±2.4×10^3^ vs 19.3±9.5×10^3^, **p=0.006, mean±SD, n=9) (figure 1D). VST fold expansion was comparable between all groups over two stimulations. After first stimulation and transduction, we achieved 48±29-fold expansion for NT, 41±36-fold expansion for TCR+, and 40±34-fold expansion for TCR8+ VSTs (mean±SD, n=6 to 8, p=ns between all groups) (figure 1E). With the second stimulation, all VSTs were further expanded for a total of 1126±979-fold expansion for NT, 736±625-fold expansion for TCR+, and 767±854-fold expansion for TCR8+ VSTs (mean±SD, p=ns between all groups) (figure 1E). Phenotypic characterization of the generated VST lines revealed a comparable subset composition with CD3+ CD4+, CD3+ CD8+ T cells, and the absence of natural killer (NK) cells (CD3− CD56+) or TCRγδ+ T cells between NT, TCR+, and TCR8+ VSTs (figure 1F). VSTs with an activated CD3+ CD56+ phenotype were increased in some donors in the TCR+ and TCR8+ lines, but the difference was not statistically significant (%CD3+ CD56+ cells in NT versus TCR+: 0±0% vs 13±15%, p=ns, NT versus TCR8+: 0±0% vs 16±19%, p=ns, TCR versus TCR8+: 13±15% vs 16±19%, p=ns, mean±SD, n=5). Memory phenotyping using the two markers CD45RO and CD62L showed a high proportion of T_CM_ cells in both, the CD8+ and the CD4+ compartment (figure 1G). In TCR8+ CD4+ VSTs, both the T_N_ and the T_CM_ compartments were better preserved compared with NT CD4+ VSTs or TCR+ CD4+ VSTs (CD4+ T_N_: NT versus TCR8+: 6.9±4.9% vs 22.0±16.8%, *p=0.03, TCR+ versus TCR8+: 3.5±2.7% vs 22.0±16.8%, **p=0.008; CD4+T_CM_: NT versus TCR8+: 54.8±7.4% vs 70.8±16.6%, *p=0.03, TCR+ versus TCR8+: 63.9±5.8% vs 70.8±16.6%, p=ns). Thus, we can reliably generate TCR and TCR8 transgenic VST lines with a predominant central memory compartment and retroviral transduction does not alter VST expansion, cellular composition or memory phenotype. Our activation, transduction and expansion process is fully GMP compatible.

### TCR8+ VSTs maintain anti-viral specificities that are comparable to NT VSTs

**Figure 2:**
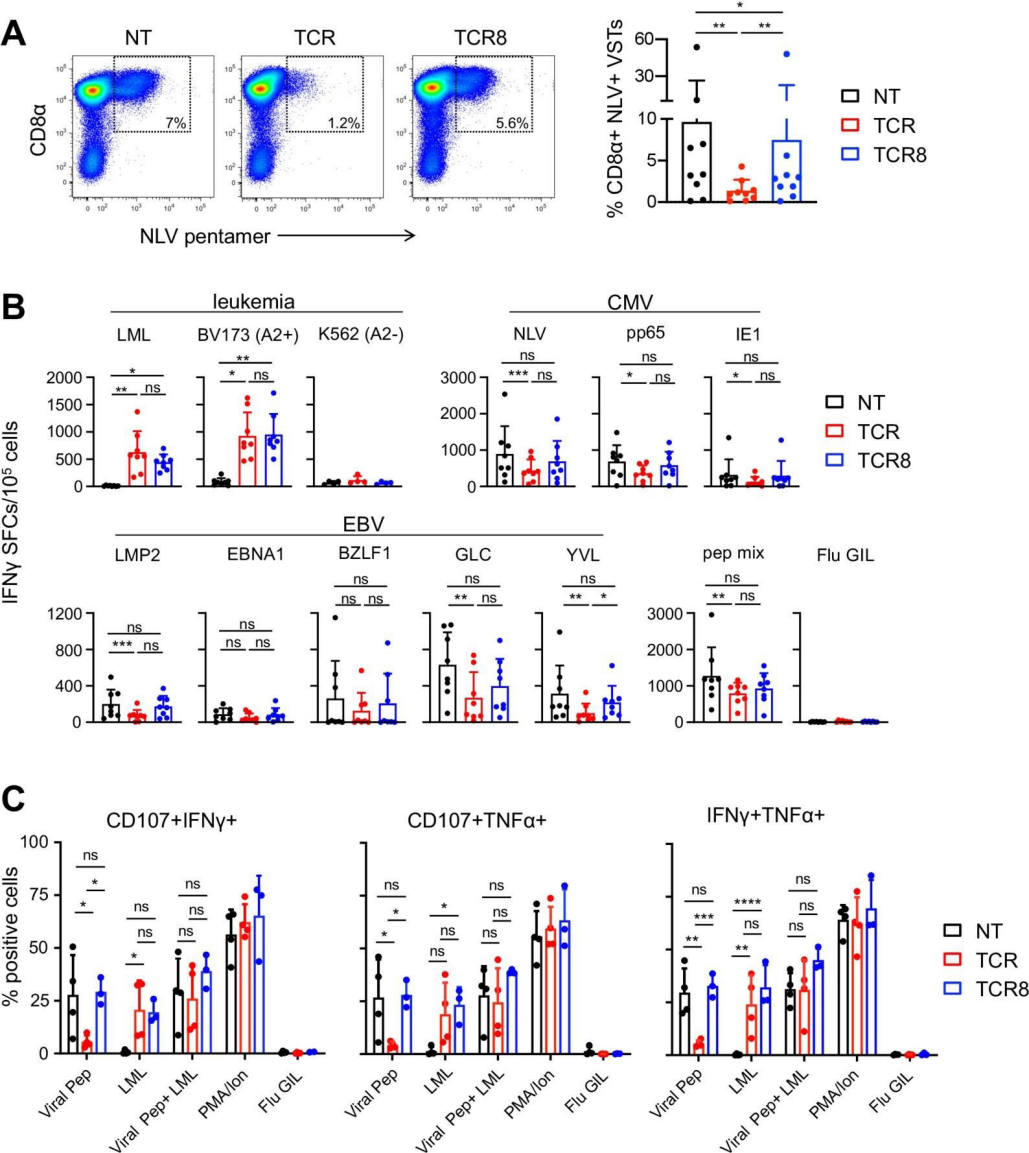
Transgenic CD8αβ is required to preserve anti-viral specificities of TCR-transgenic VSTs. (A) Frequency of CD8α+ NLV+ VSTs comparing NT, TCR+, and TCR8+ VST lines by FACS. representative FACS plots (left), summary (right), n=9, mean±SD, Wilcoxon test. (B) IFN-γ enzyme-linked immunospot comparing NT, TCR+, and TCR8+ VSTs for their anti-viral and anti-tumor (survivin) activities, n=8, mean±SD, Friedman test. Each donor was plated in technical duplicates or triplicates, dots show mean for each donor. (C) Degranulation (CD107a/b) and intracellular cytokine staining by FACS comparing NT, TCR+, and TCR8+ VSTs for their anti-viral and anti-survivin-directed reactivities. Percentage of positive cells for CD107+/IFN-γ+ (left), CD107+/TNF-α+ (middle), or IFN-γ+ TNF-α+ (right), n=4, mean±SD, two-way analysis of variance. Influenza GIL peptide: negative control. (A-C) Coding of significance levels: ns, not significant, *p<0.05, **p<0.01, ***p<0.001, ****p<0.0001. CMV, cytomegalovirus; FACS, fluorescence-activated cell sorting; IFN, interferon; NT, non-transduced; SFC, spot-forming cells; TCR, T-cell receptor; TNF, tumor necrosis factor; VSTs, virus-specific T-cells.

VST lines were evaluated for their NLV-specific pentamer staining (figure 2A) and their epitope specific IFN-γ and TNF-α production by ELISpot (figure 2B) and intracellular cytokine staining (ICS) (figure 2C) at the end of the first stimulation/expansion. NLV pentamer staining revealed a consistently and significantly reduced CD8α+ NLV+ population in TCR+ compared with NT and TCR8+ VSTs (NT versus TCR+ VSTs: 9.66±16.8% vs 1.36±1.33%, **p=0.008; TCR+ versus TCR8+ VSTs: 1.36±1.33% vs 7.48±15.31%, **p=0.008; mean±SD, n=9). The population was restored in TCR8+ VSTs to levels that were slightly but statistically significantly lower than in NT VSTs (figure 2A, NT versus TCR8+ VSTs: 9.66±16% vs 7.48±15.31%, *p=0.04). In the ELISpot and ICS, we found that TCR+ VSTs and TCR8+ VSTs produced comparable levels of IFN-γ against the cognate survivin peptide (LML) or the HLA-A*02:01+ survivin+ leukemia cell line BV173, but anti-viral reactivities against CMV (NLV peptide, pp65 and IE1 pepmixes) and EBV (GLC and YVL peptides, LMP2 pepmix) were significantly reduced in TCR+ VSTs compared with NT VSTs. We observed the rescue of cytokine production, however, in TCR8+ VSTs, which produced higher levels of IFN-γ and TNF-α than TCR+ VSTs, and comparable levels as NT VSTs (figure 2B and C). TCR8+ VSTs also degranulated in response to the stimulation with the cognate antigen as efficiently as NT VSTs, while degranulation of TCR+ VSTs was significantly reduced (figure 2C).

### Transgenic expression of CD8αβ in VSTs enhances recognition of viral epitopes

**Figure 3:**
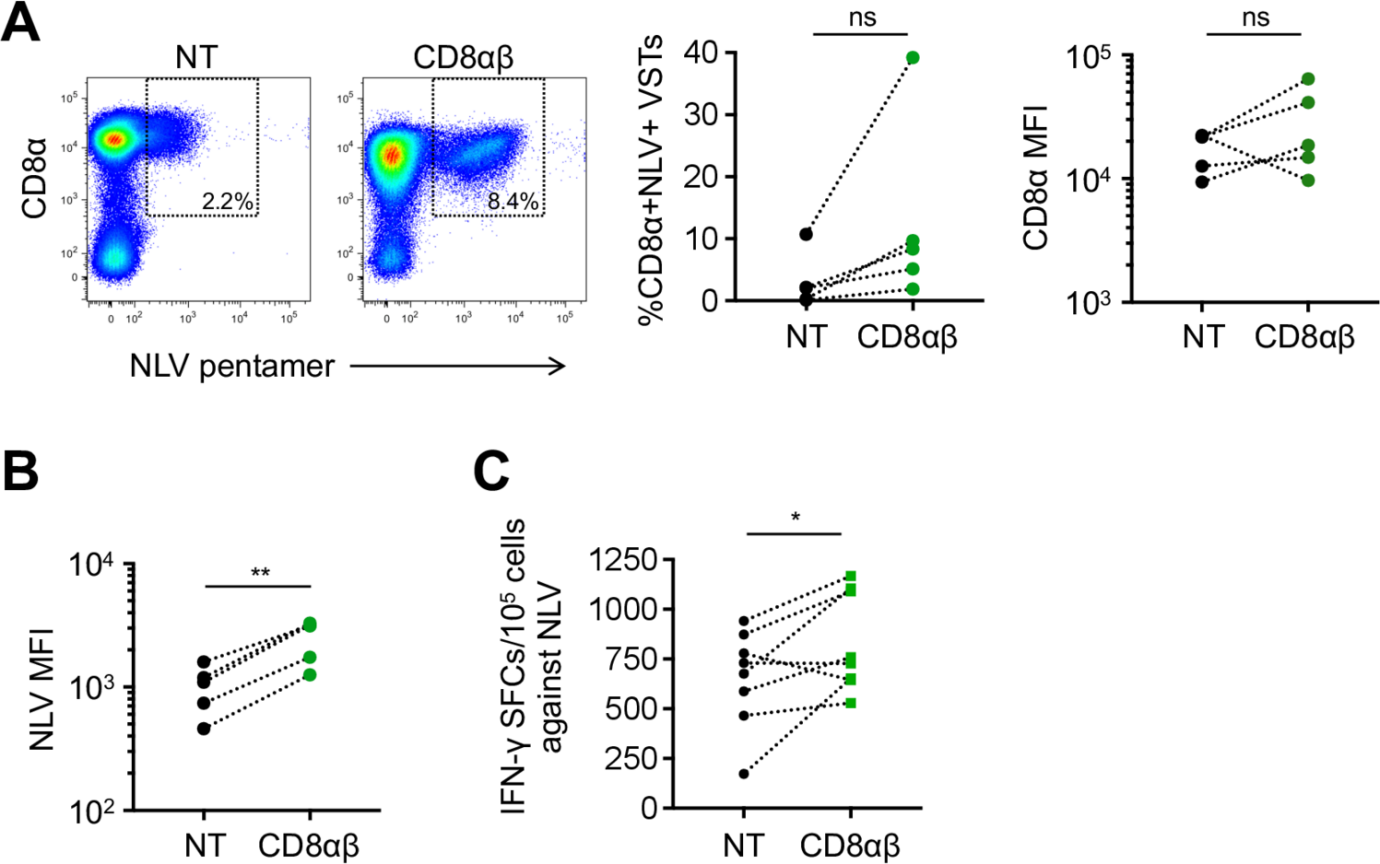
Transgenic expression of CD8αβ in VSTs enhances recognition of the NLV epitope in the absence of a transgenic TCR. (A) NLV pentamer staining of NT or CD8αβ+ transduced VSTs. Representative fluorescence-activated cell sorting plots of percentage of CD8α+ NLV+ cells (left), summary (middle), CD8α MFI (right) n=5, p=ns. (B) NLV MFI on live CD8α+ NLV+ cells, n=5. (C) IFN-γ enzyme-linked immunospot of NT or CD8αβ+ VSTs, n=3, donors plated in duplicates or triplicates, individual values shown. (A–C) Coding of significance levels: ns, not significant, *p<0.05, **p<0.01. Paired t-test. IFN, interferon; MFI, mean fluorescence intensity; NT, non-transduced; SFCs, spot-forming cells; TCR, T-cell receptor; VSTs, virus-specific T-cells.

To assess whether transgenic expression of CD8αβ enhances endogenous virus-specific TCR detection and function, we analyzed NLV pentamer staining and NLV-specific IFN-γ production of NT VSTs and CD8αβ+ VSTs. Expression of transgenic CD8αβ enhanced the detection of NLV pentamer+ VSTs at the end of the first stimulation, but was not statistically significant (%NLV+ VSTs in NT versus CD8αβ+: 3.1±4.4% versus 12.9±15%, p=ns, mean±SD, n=5) (figure 3A, left and middle panel). We also detected a trend to increased CD8α MFI in four out of five donors tested (figure 3A, right panel) but this did not reach conventional levels of significance. However, the MFI of the NLV pentamer staining was significantly higher in CD8αβ+ VSTs compared with NT VSTs (NLV MFI NT versus CD8αβ+: 1018±438 vs 2520±948, **p=0.005, mean±SD, n=5) (figure 3B). These findings are consistent with previous reports assessing the influence of CD8 co-receptor availability on major histocompatibilty complex (MHC) class I multimer stainings.^21^ With regards to NLV-specific T-cell responses determined by IFN-γ ELISpot, we found a significant increase of IFN-γ spot forming cells in CD8αβ+ VSTs compared with NT VSTs (653±246 vs 835±248, *p=0.04, mean±SD, n=3, plated in duplicates or triplicates) in response to the NLV peptide (figure 3C). Hence, forced expression of CD8αβ in VSTs enhances endogenous TCR function.

### TCR+ and TCR8+ VSTs kill viral targets in vitro

**Figure 4:**
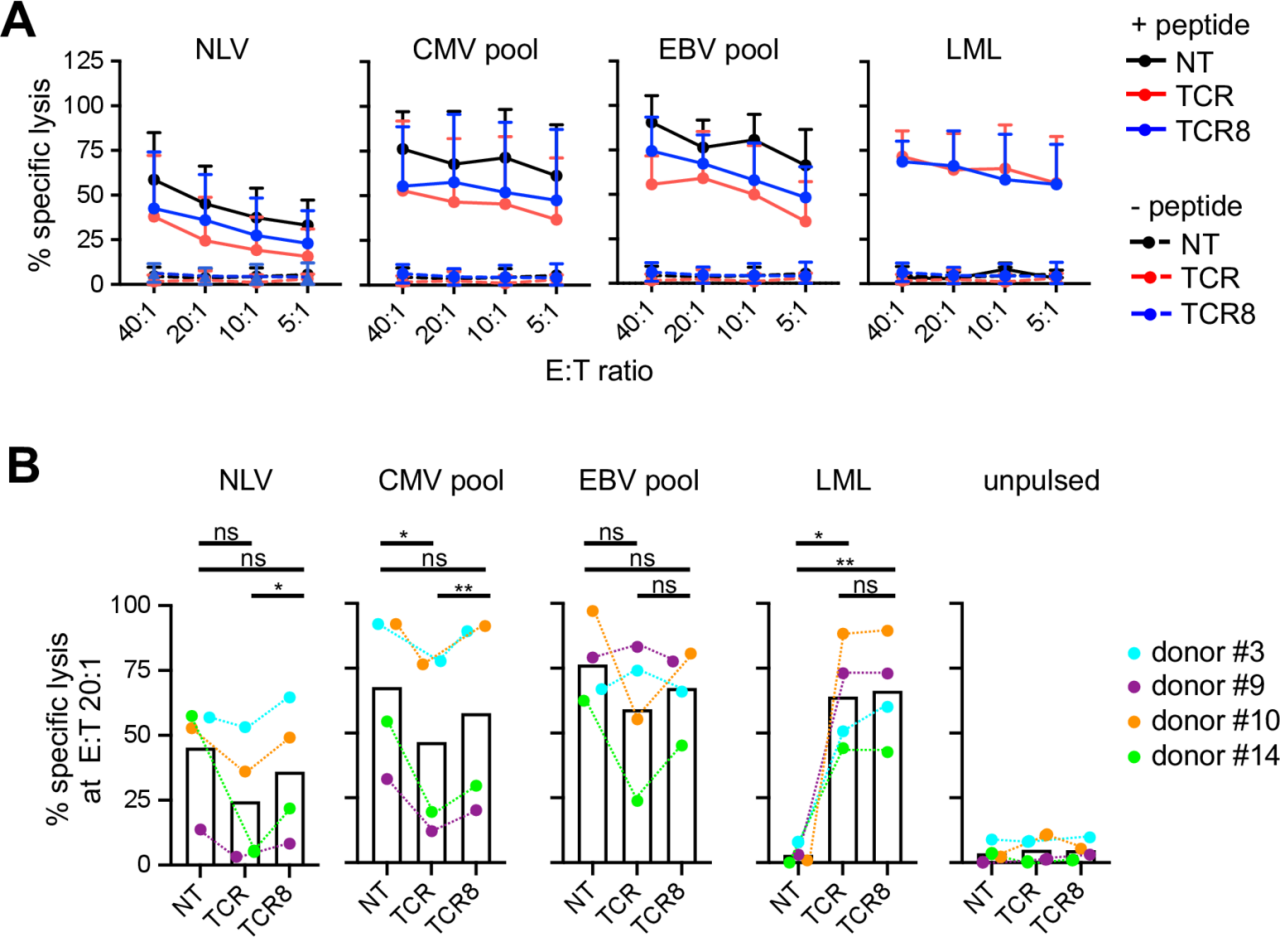
Cytotoxicity against viral targets in vitro is better preserved in VSTs engineered with TCR8 than TCR. (A) Percentage of specific lysis of peptide/pepmix pulsed or unpulsed activated autologous T cells by NT, TCR+, or TCR8+ VSTs in a 4-hour ^51^Cr-release assay at E:T ratio 40:1, 20:1, 10:1, 5:1, n=4, mean±SD. (B) Percentage of specific lysis at E:T ratio 20:1, depicting individual donors (colored lines) and average (bars). Paired t-test. ns, not significant, *p<0.05, **p<0.01. CMV, cytomegalovirus; EBV, Epstein-Barr virus; NT, non-transduced; TCR, T-cell receptor; VSTs, virus-specific T cells.

We next assessed the cytolytic activity of NT, TCR+, and TCR8+ VSTs against viral antigen-presenting target cells. In a 4-hour ^51^Cr-release assay against peptide/pepmix-pulsed activated autologous T cells, we detected specific lysis of target cells pulsed either with NLV peptide or the pooled CMV pepmixes/peptides or the pooled EBV pepmixes/peptides (solid lines), while unpulsed target cells (dashed lines) were not killed (figure 4A, mean±SD, n=4, plated in duplicates or triplicates). Survivin LML peptide pulsed targets were killed by TCR+ and TCR8+ VSTs but not NT VSTs. Overall, we observed a trend for lower cytolytic activity against viral antigen-presenting targets by TCR+ VSTs compared with TCR8+ VSTs or NT VSTs, but the overall differences were not statistically significant due to high donor variability. Donor variability is illustrated in figure 4B where we provide a summary of specific target cell lysis obtained at the E:T ratio of 20:1, depicting individual dots per donor and average bars. Some donors lysed targets to a similar extent with TCR+ or TCR8+ VSTs (eg, donor #3, cyan), while others showed a significant drop when comparing NT versus TCR+ VSTs, and a partial restoration of the activity with TCR8+ VSTs (eg, donor #14, green). Overall, we found a significant enhancement of CMV-specific target cell lysis (both NLV peptide and CMV pool) with TCR8+ VSTs compared with TCR+ VSTs, while the analysis for the EBV pool did not reach significance. One donor had a weak baseline CMV and a strong EBV response (donor #9, purple), while the others had strong baseline responses against both viruses. Killing of LML peptide-pulsed target cells was consistent with both TCR+ VSTs and TCR8+ VSTs, and unpulsed targets were not killed.

### Anti-leukemic activity of TCR+ and TCR8+ VSTs in vitro and in vivo

**Figure 5:**
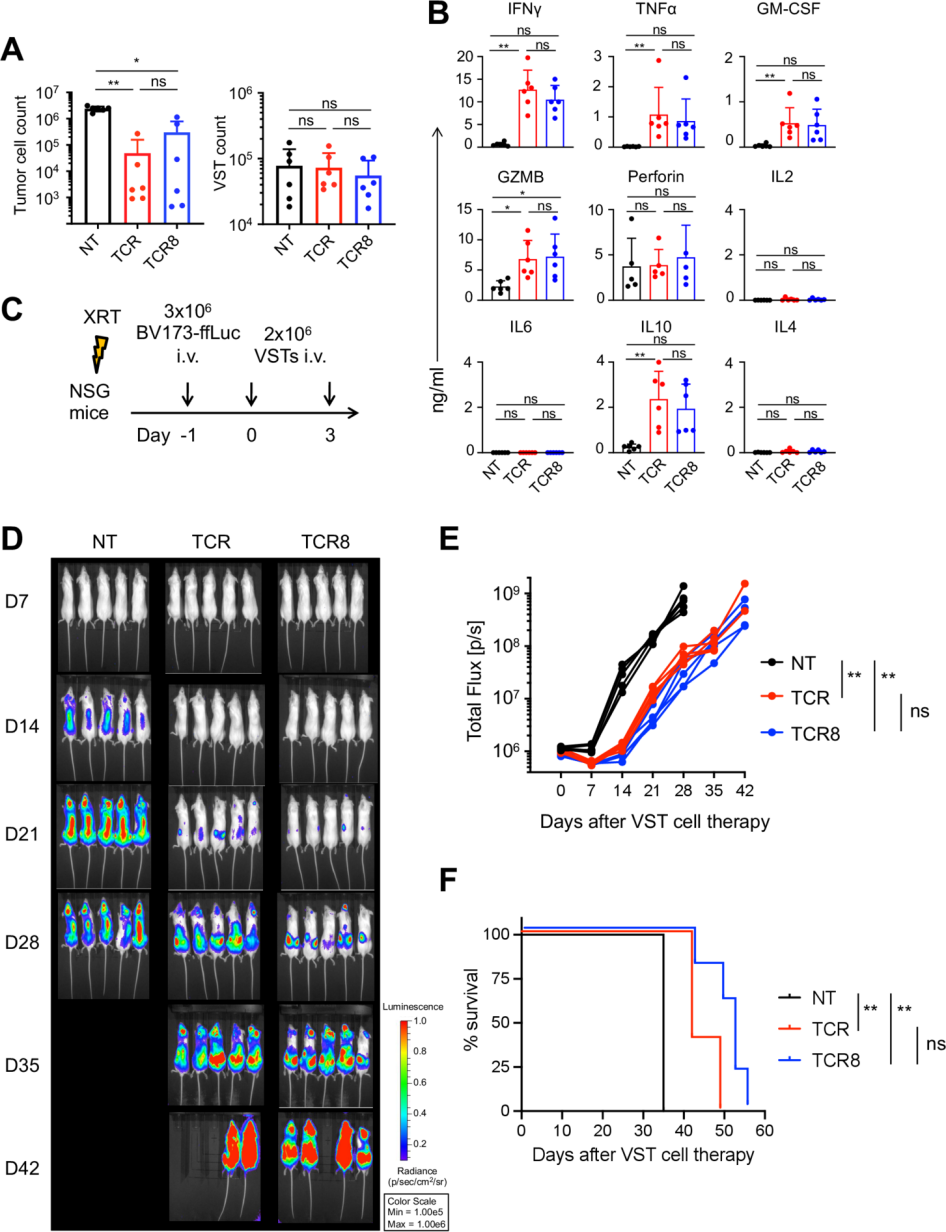
TCR+ and TCR8+ VSTs kill leukemia in vitro and in vivo and produce T_H_1 cytokines. (A) Co-culture of NT, TCR+, or TCR8+ VSTs with BV173 leukemia cells (HLA-A*02:01+ survivin+); E:T ratio 1:5. residual BV173 (left) or VSTs (right) were quantified by fluorescence-activated cell sorting on day 3, n=6, mean±SD, one-way analysis of variance on log transformed data. (B) Cytokine production after 24 hours in co-culture, n=6, mean±SD, Friedman test. (C) Mouse xenograft model experimental set up. (D) Individual mouse pictures of BLI, color scale 1×10^5^ to 1×10^6^ photons/s/cm^2^/sr. (E) Summary of BLI data from mice treated with control (NT, black), TCR+ (red), or TCR8+ (blue) VSTs. n=5 mice per group, individual lines per mouse. Mann-Whitney test on area under the curves from day 0 to 28. (F) Kaplan-Meier survival analysis of mice treated with control (NT, black), TCR+ (red), or TCR8+ (blue) VSTs. n=5 mice per group, log-rank test. (A–F) Coding of significance levels: ns, not significant, *p<0.05, **p<0.01, ***p<0.001, ****p<0.0001. BLI, bioluminescent imaging; GM-CSF, granulocyte-macrophage colony-stimulating factor; IFN, interferon; IL, interleukin; TCR, T-cell receptor; TNF, tumor necrosis factor; NT, non-transduced; VSTs, virus-specific T cells.

We next assessed the anti-leukemic activity of TCR+ and TCR8+ VSTs compared with NT VST controls in a co-culture assay with BV173 leukemia cells (HLA-A*02:01+ survivin+). We found significant cytotoxicity with TCR+ and TCR8+ VSTs by quantification of residual tumor cells in culture after 3 days (tumor cell count NT versus TCR+: 2.4±0.5×10^6^ versus 0.04±0.1×10^6^, **p=0.004, NT versus TCR8+: 2.4±0.5×10^6^ versus 0.3±0.4×10^6^, *p=0.04, TCR+ versus TCR8+: 0.04±0.1×10^6^ vs 0.3±0.4×10^6^, p=ns, mean±SD, n=6) (figure 5A). VST counts at the end of the co-cultures were not different between groups. Analysis of co-culture supernatants collected 24 hours after initial tumor challenge revealed that both TCR+ and TCR8+ VSTs produced significant amounts of T_H_1 cytokines (IFN-γ NT versus TCR+: 0.6±0.4 vs 12.7±4.3 ng/mL, **p=0.007; NT versus TCR8+: 0.6±0.4 vs 10.5±3.1 ng/mL, p=ns; TCR+ versus TCR8+, p=ns, TNF-α NT versus TCR+: 0.02±0.008 vs 1.0±0.9 ng/mL, **p=0.005, NT versus TCR8+: 0.02±0.008 vs 0.9±0.7 ng/mL, p=ns, TCR+ versus TCR8+, p=ns, mean±SD, n=6) and cytotoxic granules (Granzyme B (GZMB) NT versus TCR+: 2.3±0.9 vs 6.8±4.3 ng/mL, *p=0.03, NT versus TCR8+: 2.3±0.9 vs 7.2±3.7 ng/mL, *p=0.03, TCR+ versus TCR8+: p=ns, mean±SD, n=6) (figure 5B). We also detected cytotoxic granule release into the supernatant of NT VSTs even though no (non-specific) killing activity was detectable. Low levels of IL10 production were detected from TCR+ and TCR8+ VSTs (figure 5B). To assess the anti-leukemic function of engineered VSTs in vivo, we used our previously established NSG xenograft model with BV173-ffLuc cells (figure 5C). One day after leukemia challenge, the mice received two infusions of NT, TCR+, or TCR8+ VSTs 3 days apart and tumor growth was assessed over time by BLI. Mice treated with TCR+ and TCR8+ VSTs experienced significantly slower leukemia progression than mice treated with NT VSTs, indicating that both TCR+ and TCR8+ VSTs produced significant anti-leukemic activity in mice (AUC on day 28: NT versus TCR+: **p=0.008, NT versus TCR8+: **p=0.008, TCR+ versus TCR8+: p=0.05, ns, n=5 mice per group) (figure 5D and E). There was also a trend to better leukemia control by TCR8+ VSTs compared with TCR+ VSTs, although not statistically significant (p=0.05). The anti-leukemic activity of TCR+ VSTs and TCR8+ VSTs also significantly improved the survival of mice (NT versus TCR+: **p=0.003, NT versus TCR8+: **p=0.003, TCR+ versus TCR8+: p=0.05, ns, n=5 mice per group), with again a trend to better survival with TCR8+ VSTs (figure 5F).

## Discussion

We show that transgenic CD8αβ alone or in combination with a transgenic TCR promotes endogenous TCR function in VSTs expressing a second transgenic TCR. TCR8 expression in VSTs rescues endogenous anti-viral activity to levels comparable to non-transduced VSTs while maintaining anti-tumor function mediated by the transgenic TCR. These benefits are observed both in vitro and in vivo. Thus, our approach overcomes the competition for essential signaling components that leads to downregulation of anti-viral reactivities and impedes full therapeutic benefit of providing anti-viral and anti-tumor function through endogenous and transgenic TCRs.

The concept of providing simultaneous anti-viral and anti-tumor function in one cell therapy product was clinically validated when autologous CAR modified VSTs were shown to be able to target both EBV and GD2 in patients with neuroblastoma.^2^ After adoptive transfer, GD2-CAR+ EBVSTs demonstrated safety, anti-tumor function, and long-term persistence in patients.^2 3^ Since then, the approach was extended to CD19-CARs expressed in allogeneic stem cell donor-derived VSTs and infused to children with high risk B-cell acute lymphoblastic leukemia in remission after allogeneic HSCT,^4–6^ in whom in vivo expansion of CD19-CAR+ VSTs during viral reactivation was observed.^6^ Similarly, vaccination of subjects with varicella zoster virus may be another approach that augments expansion of VSTs expressing a GD2 CAR in patients with sarcoma or neuroblastoma (NCT01953900).^6^ Thus, in principle, stimulation through the endogenous virus-specific TCR and co-stimulation on antigen-presenting cells in vivo can produce significant re-expansion and function of infused CAR+ VSTs.

It is more challenging to extend the VST platform to target tumor antigens by means of transgenic TCRs rather than CARs. Endogenous and transgenic TCRs compete for the same TCR signaling complex components (including CD3 chains and the CD8αβ co-receptor), and introduced TCR chains may mis-pair with endogenous TCR chains, leading to an overall reduced anti-viral activity.^7–12^ The use of murine-constant regions in the transgenic TCR can significantly reduce mis-pairing.^13^ Transfer of CD8αβ can redirect CD4+ T cells to class I-restricted antigens, and the transgenic CD4+ T cells become cytotoxic hybrid cells.^14 22–27^ We have recently analyzed the transcriptional consequences of TCR8 expression in polyclonal CD4+ and CD8+ T cells by single cell RNA sequencing and experimental validation and showed that transgenic TCR8 has multiple advantages in both CD4+ and CD8+ lineages, promoting an overall enhanced anti-tumor function.^14^ We now investigated the impact of transgenic CD8αβ on endogenous class I-restricted TCR function in CD8+ T cells as we hypothesized that combining CD8αβ co-receptor as a transgene with a tumor-targeted TCR is one potential means of overcoming the limitation of reduced anti-viral activity in TCR+ VSTs. We first confirmed that anti-viral specificity and activity was reduced when our survivin-specific TCR is expressed in VSTs. Next, we demonstrated that TCR8 rescued endogenous anti-viral TCR specificity and activity, using pentamer staining, IFN-γ ELISpot, degranulation assay and ICS, as well as cytotoxicity assays. TCR8+ VSTs retained specificity and anti-viral activity that was comparable to NT VSTs. We also showed that the transgenic CD8αβ effect on endogenous TCR function was preserved in vitro even in the absence of a transgenic TCR. These findings indicate that (i) TCR8+ VSTs respond to viral infections to a comparable level as NT VSTs, and (ii) TCR8+ VSTs are ready to re-expand on viral challenge. Importantly, the in vivo anti-tumor function of TCR8+ VSTs was retained. However, the in vivo anti-viral activity of TCR8+ VSTs remains to be validated, an analysis that will be a component of ongoing and proposed human studies.^2–4 6^ Several features of the CD8αβ co-receptor most likely explain our findings, including its role in TCR-pMHC recognition and modulation of antigen-sensitivity,^28^ recruitment of Lck to the immune synapse, and activation of signaling components that are crucial during early T-cell activation,^29^ and, as recently demonstrated by single cell RNA sequencing, its impact on a variety of transcriptional pathways upon tumor challenge that support enhanced anti-tumor function.^14^

An additional advantage of VSTs as a platform for the delivery of tumor-antigen-specific T-cell function to treat cancer patients lies in the fact that transgenic VSTs can be generated from well-characterized healthy donors for allogeneic cell banks. Indeed, third party donor allogeneic ‘off-the-shelf’ banked VSTs have been clinically validated for the treatment of viral infections or EBV-associated lymphoproliferation in immunocompromised patients after HSCT or solid organ transplant.^18 30–33^ Third party donor VSTs could be engineered with TCRs or CARs, banked, and thereby provide a readily available ‘off-the shelf’ product that does not require genome editing for safe infusion in immunocompromised patients.^34^ Furthermore, additional engineering can be incorporated to protect VSTs from host NK and/or T-cell mediated allograft rejection, furthering their potential to become a safe universal ‘off-the shelf’ T-cell product with long in vivo persistence.^35 36^

Stimulation of T cells with viral peptide-pulsed autologous DCs followed by retroviral transduction yielded TCR+ or TCR8+ VST lines consistently composed of CD4+ and CD8+ VSTs with a predominant central memory phenotype and high transduction efficiencies. TCR8 expression was associated with a better preservation of less differentiated VSTs in the CD4+ compartment.^14^ The only HLA restriction of the product is defined by the transgenic TCR used to redirect VSTs to the cancer. Feasibility and safety of our manufacturing have already been demonstrated in CAR+ VST clinical trials and are GMP compliant.^2–4 6^

In summary, we show that transgenic CD8αβ rescues endogenous anti-viral TCR function in VSTs when combined with a tumor-targeted transgenic TCR. Our TCR8+ VST product provides significant anti-tumor function in vivo. We propose that the use of a combined TCR8 vector may better maintain in vivo long-term benefits of VSTs than cells that lack this modification. Our clinically validated VST platform is amenable to genetic engineering with TCR8 and ready to clinically assess the safety and efficacy to prevent and treat viral infection and malignant relapse after allogeneic HSCT.
